# Efficacy of weekly paclitaxel for the treatment of advanced ovarian cancer: A protocol for systematic review and meta-analysis

**DOI:** 10.1097/MD.0000000000020537

**Published:** 2020-06-19

**Authors:** Dong-xu Zhao, Ping Chen, Cui-hong Su, Yan-yan Zhao, Li-dan Sun, Hong He, Xiao-na Feng

**Affiliations:** a Department of Obstetrics and Gynecology, First Affiliated Hospital of Jiamusi University, Jiamusi; b Department of Obstetrics and Gynecology, Shenzhen Sami Medical Center, Shenzhen, China.

**Keywords:** advanced ovarian cancer, efficacy, safety, weekly paclitaxel

## Abstract

**Background:**

This study aims to assess the efficacy and safety of weekly paclitaxel (WP) for the treatment of advanced ovarian cancer (AOC).

**Methods:**

This study will systematically search bibliographic databases (MEDLINE, EMBASE, Cochrane Library, Web of Science, CINAHL, PSYCINFO, Allied and Complementary Medicine Database, CNKI, WANGFANG, and Chinese Biomedical Literature Database) and other literature sources from inception to the March 1, 2020 without language and publication time limitations. Two authors will independently complete all literature selection, data collection, and study quality evaluation. Any disagreements will be solved by a third author through discussion. We will analyze data by RevMan V.5.3 software.

**Results:**

This study will systematically generate a comprehensive summary on the efficacy and safety of WP for the treatment of AOC.

**Conclusion:**

This study may provide beneficial evidence of WP for the treatment of AOC.

**Systematic review registration:**

INPLASY202040193.

## 1 Introduction

Ovarian cancer (OC) is 1 of the most lethal gynecologic cancers, and also the leading cause of cancer-related deaths.^[1–2]^ Despite the research of CC achieved greatly during the past few decades, almost 70% of the patients relapse, and developed to advanced ovarian cancer (AOC).^[3–7]^ Thus, effective therapy schedule is very important to treat patients with AOC, such as weekly paclitaxel (WP).^[8–12]^ Although many studies have assessed the efficacy and safety of WP for the treatment of patients with AOC,^[11–25]^ no systematic review has been done on this topic. Therefore, this study will include only gather data from eligible randomized controlled trials (RCTs) to provide further knowledge on the efficacy and safety of WP for the treatment of AOC.

## 2 Methods

### 2.1 Study registration

This protocol has been registered on INPLASY202040193. We report it based on the guidelines of the Preferred Reporting Items for Systematic Reviews and Meta-Analysis Protocol statement.^[26–27]^

### 2.2 Ethics and dissemination

This study will not need ethical approval, since no personal patient data will be used. This study is expected to be published on a peer-reviewed journal.

### 2.3 Eligibility criteria for study selection

#### 2.3.1 Type of studies

This study will include RCTs that assessed the efficacy and safety of WP for the treatment of AOC. We will exclude other studies, such as animal studies, reviews, comments, case studies, non-controlled trials, and non-RCTs.

#### 2.3.2 Type of participants

This study will include any patients who were diagnosed as AOC, irrespective nationality, race, sex, and economic status.

#### 2.3.3 Type of interventions

In the interventional group, all patients who received WP will be included as their therapy.

In the control group, all patients who could receive any treatments will be included as their comparator.

#### 2.3.4 Type of outcome measurements

Outcomes are overall survival, pathological complete response, cancer-specific survival, recurrence-free survival, disease-free survival, and adverse events.

### 2.4 Data sources and search strategy

Two authors will perform systematic and comprehensive literature sources in bibliographic databases (MEDLINE, EMBASE, Cochrane Library, Web of Science, CINAHL, PSYCINFO, Allied and Complementary Medicine Database, CNKI, WANGFANG, and Chinese Biomedical Literature Database) from inception to the March 1, 2020 without language and publication time restrictions. We present search strategy of MEDLINE in Table 1, and will adapt similar search strategies for other electronic databases. In addition, we will identify other literature sources from dissertations, thesis, conference abstracts, and reference lists of relevant reviews.

**Table 1:**
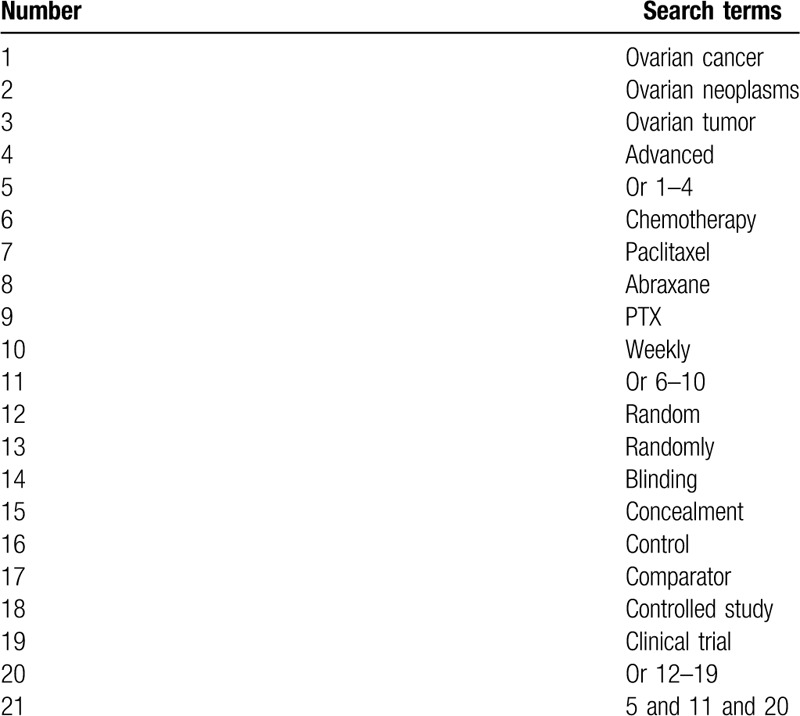
Search strategy for MEDLINE.

### 2.5 Data collection and management

#### 2.5.1 Study selection

Two authors will independently screen the titles/abstracts of all searched literatures, and all unconnected studies will be eliminated. The full papers of all potential studies will be obtained to further identify them against inclusion criteria. If any disagreements between 2 authors occur, a third author will solve them through discussion, and a final decision will be reached. We will present results of study selection in a flowchart.

#### 2.5.2 Data extraction

Two authors will conduct data extraction using a standardized data collection. Any conflicts between 2 authors will be resolved by a third author via discussion. The extracted information includes publication information (eg, title, first author, year of publication), participant information (eg, gender, age, and eligibility criteria), study methods, details of treatments and controls (eg, types of interventions, dosage, and frequency), outcome indicators, results, and conclusions.

If we identify any unclear or missing data, we will contact primary authors to request those data. If we can not obtain such data, we will utilize and analyze available data only.

### 2.6 Risk of bias assessment

Two authors will use Cochrane risk of bias tool to assess risk of bias for each included trial, respectively. It includes 7 items and each 1 is rated as high, unclear, and low risk of bias. Any discrepancies will be solved through discussion with the help of a third author.

### 2.7 Treatment effect measurements

We will express continuous outcomes as mean differences or standardized mean differences and 95% confidence intervals (CIs), and dichotomous outcomes as risk ratios or odds ratios and 95% CIs.

### 2.8 Statistical analysis

This study will utilize RevMan V.5.3 software to synthesize and analyze the data. *I*^*2*^ statistic test will be used to check heterogeneity across included trials. *I*^*2*^ ≤50% means acceptable heterogeneity, and we will use a fixed-effect model to pool the data, and to conduct a meta-analysis. On the other hand, *I*^*2*^ >50% indicates obvious heterogeneity, and we will use a random-effect model to synthesize data. In addition, we will undertake subgroup analysis and sensitivity analysis to explore the sources of remarkable heterogeneity. If necessary, a narrative summary will be conducted to report merged outcome results.

Subgroup analysis will be carried out according to the different study information, treatments, controls and outcomes. Sensitivity analysis will be performed to test the robustness of study findings by eliminating low quality trials. Reporting bias will be examined by funnel plots and Egger linear regression test if over 10 trials are included.^[28–29]^

## 3 Discussion

Recent clinical studies have indicated that WP might benefit for patients with AOC.^[11–25]^ Up to now, there is no published systematic review on WP for the treatment of AOC. This study will synthesize relevant data comprehensively and systematically to reflect the integrated efficacy and safety of the eligible trials. The results of this study may provide high-quality evidence-based medicine evidence to determine whether WP is effective and safe for the treatment of patients with AOC or not.

## Author contributions

**Conceptualization:** Dong-xu Zhao, Ping Chen, Yan-yan Zhao, Hong He, Xiao-na Feng.

**Data curation:** Cui-hong Su, Hong He.

**Formal analysis:** Dong-xu Zhao, Ping Chen, Yan-yan Zhao, Li-dan Sun.

**Funding acquisition:** Xiao-na Feng.

**Investigation:** Xiao-na Feng.

**Methodology:** Dong-xu Zhao, Ping Chen, Cui-hong Su, Li-dan Sun, Hong He.

**Project administration:** Xiao-na Feng.

**Resources:** Dong-xu Zhao, Yan-yan Zhao, Li-dan Sun, Hong He.

**Software:** Dong-xu Zhao, Cui-hong Su, Yan-yan Zhao, Li-dan Sun, Hong He.

**Supervision:** Xiao-na Feng.

**Validation:** Dong-xu Zhao, Yan-yan Zhao, Li-dan Sun, Hong He, Xiao-na Feng.

**Visualization:** Dong-xu Zhao, Ping Chen, Cui-hong Su, Li-dan Sun, Xiao-na Feng.

**Writing – original draft:** Dong-xu Zhao, Ping Chen, Yan-yan Zhao, Xiao-na Feng.

**Writing – review & editing:** Dong-xu Zhao, Cui-hong Su, Yan-yan Zhao, Li-dan Sun, Hong He, Xiao-na Feng.
